# Effect of Delayed Surgery for Ventricular Septal Rupture on Postoperative Outcomes

**DOI:** 10.7759/cureus.66655

**Published:** 2024-08-11

**Authors:** Kohei Sumi, Tomohiro Iwakura, Ryangwon Yoon, Yoshinori Nakahara, Masanari Kuwabara, Akira Marui

**Affiliations:** 1 Department of Cardiothoracic Surgery, Sakakibara Heart Institute, Tokyo, JPN; 2 Department of Cardiology, Toranomon Hospital, Tokyo, JPN

**Keywords:** coronary artery bypass grafting (cabg), mechanical circulatory assistant devices, extracorporeal membrane oxygenation support, acute myocardial infarction, post-myocardial infarction ventricular septal rupture

## Abstract

Objectives: The prognosis of ventricular septal rupture (VSR) after acute myocardial infarction remains poor; hence, surgical repair is essential. However, the appropriate timing for surgical intervention remains unclear. We aimed to compare the prognosis between early (<96 hours) and delayed (≥96 hours) surgery for VSR.

Methods: This single-center, retrospective cohort study used data from 49 patients who underwent VSR repair after acute myocardial infarction (AMI) between 2007 and 2022 at our institution. In-hospital and one-, three-, and 10-year mortality and major adverse cardiac and cerebrovascular events were compared between the early (group A) and delayed (group B) surgery after AMI.

Results: No significant differences were found between the patients’ backgrounds of the two groups. The in-hospital mortality rates were 37.5 and 16.0% for groups A and B, respectively (*P* = 0.114). The overall survival rates estimated using Kaplan-Meier analysis were 66.5 ± 6.9, 58.2 ± 7.5, and 28.8 ± 10.6% after one, three, and 10 years, respectively. The mortality rates in group B at three (hazard risk ratio: 2.691; 95% confidence interval: 1.02-7.097) and 10 (hazard risk ratio: 2.575; 95% confidence interval: 1.125-5.891) years were significantly better than those in group A. Major adverse cardiac and cerebrovascular events were significantly different between the two groups at all time points.

Conclusions: These results showed that patients who underwent surgery for VSR 96 hours after AMI had better long-term survival than those who underwent surgery within 96 hours.

## Introduction

Ventricular septal rupture (VSR) represents a critical mechanical complication following acute myocardial infarction (AMI) and continues to carry a grave prognosis despite advancements in surgical techniques [1-4]. The standard of care for VSR repair has evolved alongside improvements in medical therapies such as mechanical circulatory support, intra-aortic balloon pumps (IABP), and extracorporeal membrane oxygenation (ECMO), which have enabled a more tailored approach to the timing of surgical intervention [5].

Surgical repair of VSR can be delayed to allow for the optimal healing of myocardial tissue, potentially enhancing the stability of repair. Delayed intervention aims to capitalize on the progression of fibrosis in the infarcted myocardium, thereby improving surgical outcomes [5].

Despite these advancements, there is a lack of consensus and comprehensive data on how the timing of VSR repair affects long-term outcomes, such as survival rates and major adverse cardiac and cerebrovascular events (MACCE). The current literature provides limited insight into the optimal timing for surgical intervention and its impact on patient prognosis.

This study aims to explore the benefits and risks associated with delaying surgical repair of post-AMI VSR, comparing outcomes between patients who underwent early (<96 hours) versus delayed (≥96 hours) surgery. By investigating these outcomes, we seek to provide insights into the optimal timing of surgical intervention for VSR, thereby contributing to improving patient care.

## Materials and methods

Ethical statement

This study adhered to the principles of the Declaration of Helsinki. The study was approved by the Sakakibara Heart Institute Institutional Ethics Committee (clinical registration number: 22-034) and consent was obtained from all participants using a comprehensive agreement provided by the Sakakibara Heart Institute. All data were collected and compiled using a secure computer database, and individual data were anonymized without identifiable personal information. 

Study participants and outcome measurement

From April 2007 to July 2022, 49 consecutive patients who were diagnosed with post-AMI VSR underwent surgical repair at Sakakibara Heart Institute, Fuchu, Tokyo, Japan. Patient data were collected based on medical records and analyzed retrospectively. The majority of cases were referral patients from other medical institutions and were admitted to our hospital with already diagnosed VSR. Consequently, attempting to distinguish between the onset of VSR and AMI during medical history assessment could yield potentially inaccurate data. Hence, for this study, data collection was standardized to the day of AMI onset.

Patient selection and grouping

**Figure 1:**
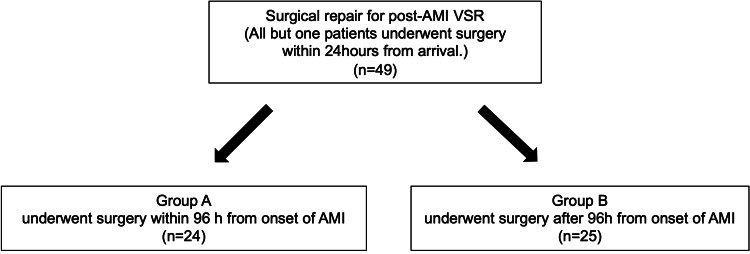
Flow chart for the study Forty-nine (49) patients were analysed with post-acute myocardial infarction (AMI) ventricular septal rupture (VSR) divided into two groups based on the time of VSR repair from the onset of AMI: A (n = 24, <96 hours) and B (n = 25, ≥96 hours, with a maximum delay of 43 days).

All patients with suspected VSR were evaluated using coronary angiography, regardless of whether they had undergone coronary angiography or percutaneous coronary intervention elsewhere before referral. In principle, an IABP was inserted preoperatively, and ECMO was considered when cardiogenic shock was observed. Surgery was performed upon admission at our hospital following preoperative examinations and the establishment of circulatory support. All but one patient underwent surgery within 24 hours of arrival at our hospital. Patients who underwent surgery within 96 hours and those who underwent surgery ≥96 hours after the onset of AMI were retrospectively assigned to the early (group A, n = 24) and delayed (group B, n = 25) groups, respectively (Figure 1). The reason for the 96-hour stratification is twofold: first, for an equal distribution of the number of cases, and second, based on studies indicating favorable outcomes in cases with surgical repair of post-AMI VSR operated on from day 4 after the diagnosis of VSR, compared to earlier interventions [6].

Outcome measures

The early postoperative outcomes compared were in-hospital or 30-day mortality and postoperative complications, including prolonged ventilation, tracheostomy, postoperative stroke, reoperation, recurrent or residual shunt, re-exploration for bleeding, severe arrhythmia, postoperative atrial fibrillation, renal replacement therapy, and deep sternal wound infection. The patients were followed up after discharge and contacted by physicians for details of any outcome event. Long-term clinical outcomes compared between the two groups were all-cause death and major adverse cardiac and cerebrovascular events (MACCE). MACCE included death, stroke, and myocardial infarction.

Surgical procedures

From 2007 to 2015, VSR repair was performed using the modified David-Komeda infarction exclusion method [7]. From 2016 onwards, the extended sandwich patch technique [8,9] has been used for VSR repair. 

Modified David-Komeda Infarction Exclusion Technique

This modified method is similar to that described by Sugimoto et al. [10]. A left ventriculostomy, parallel to and 2 cm distant from the left anterior descending artery, was performed through the infarcted anterior wall. The VSR defects were closed directly using a tailored small bovine pericardial patch. Then, two large rectangular-shaped bovine pericardial patches were prepared: one was sutured to the non-infarcted endocardium around the ventricular septal side, and the other was sutured to the anterolateral ventricular wall. This suture was transmural and reinforced with a polytetrafluoroethylene felt strip placed outside the left ventricular wall. The two bovine pericardial patches were then properly cut and sewn into each other. These patches were shaped like pouches to fit the left ventricular cavity and exclude the infarcted muscle. Bio Glue®︎ (Cryolife Inc, Kennesaw, Ga) and SURGICEL®︎ FIBRILLAR™️ (Ethicon, USA) were used to fill the cavity between the patch and ventricular wall, and the ventriculostomy was closed in two layers using polytetrafluoroethylene felt strips.

Extended Sandwich Patch Technique

Right ventriculostomy was performed parallel to and 1 cm distant from the left anterior descending artery. The infarcted lesion was inspected, and the necrotic muscle’s margin was resected to pass a patch through the defect into the left ventricle. Two octagonal bovine pericardial patches were tailored to be large enough to overlap the defect margin by ≥1.5 cm. The first patch was passed through the right ventricle and attached to the left ventricular side of the septal wall. The second patch was attached to the right ventricular side of the septal wall. Subsequently, the defect was sandwiched between the two patches. Bio Glue®︎ and SURGICEL®︎ FIBRILLAR™️ were used to fill the defect, and the ventriculostomy was closed with polytetrafluoroethylene felt strips.

After VSR repair, concomitant procedures were performed. Coronary artery bypass grafting was performed on the non-culprit vessels with significant stenosis.

Preoperative and postoperative care

Preoperative care included a standard assessment of cardiac function and localization of VSR defect by echocardiography. Mechanical support, such as IABP or ECMO, was provided as needed. Postoperative care involves monitoring urine output and vital signs. Typically, IABP and ECMO support was maintained for three to four days. Extubation was considered when vital signs, including urine output were stable, and the patient was alert and oriented.

Statistical analysis

Categorical variables are reported as the number and percentage of patients, and the between-group differences in these variables were analyzed using chi-square or Fisher's exact tests. Continuous variables are reported as mean ± standard deviation (SD), with between-group differences being analyzed using Student's t-test or the Mann-Whitney U test. Statistical significance was set at P < 0.05. Kaplan-Meier curves were used to analyze survival and MACCE; they were plotted for up to 10 years of follow-up and compared using the log-rank test. Cox proportional hazards analysis was performed to evaluate the hazard risk ratio and 95% confidence interval. In addition, hazard risk ratio was evaluated using crude models and multivariate logistic regression models with adjustments for (i) EuroSCORE II (age, sex, renal impairment, extracardiac arteriopathy, poor mobility, previous cardiac surgery, pulmonary disease, active endocarditis, critical preoperative state, diabetes on insulin, Canadian Cardiovascular Society functional classification angina class 4, New York Heart Association class, left ventricular ejection fraction, recent myocardial infarction, pulmonary artery pressure, and the urgency and weight of the procedure) [11]; (ii) preoperative cardiopulmonary resuscitation; and (iii) preoperative ECMO. Statistical analysis was performed using R 4.1.1 (R Project for Statistical Computing, R Foundation, Vienna, Austria) and EZR (Saitama Medical Center, Jichi Medical University, Saitama, Japan). 

## Results

Patient background

The baseline patient characteristics and intraoperative data are presented in Table 1. No significant differences in patient backgrounds were observed between groups A and B, except for the duration from AMI to surgery (P < 0.001). The mean operation, cardiopulmonary bypass, cross-clamp times, and number of concomitant surgeries in each group were not significantly different between the groups.

**Table 1:** Baseline patient characteristics (n = 49) Data are presented as n (%) unless otherwise indicated. P values less than 0.05 were considered statistically significant. AMI, acute myocardial infarction; BSA, body surface area; COPD, chronic obstructive pulmonary disease; CPB, cardiopulmonary bypass; Cr, creatinine; IABP, Intra-aortic balloon pump; ECMO, extracorporeal membrane oxygenation; PCI, percutaneous coronary intervention; SD, standard deviation; VSR, ventricular septal rupture. *a* Determined by Student t-test, *b* Determined by Chi-square test, *c* Determined by Mann–Whitney U test, *d* Determined by Fisher's exact tests

	All n = 49	Group A n = 24	Group B n = 25	P-value
Age (years, mean ± SD)	75.0 ± 9.6	76.6 ± 8.0	73.4 ± 10.9	0.24^a^
Female	18 (36.7)	10 (41.7)	8 (32.0)	0.56^b^
BSA (m^2^, mean ± SD)	1.60 ± 0.19	1.58 ± 0.20	1.61 ± 0.18	0.58^a^
Hypertension	29 (59.2)	16 (66.7)	13 (52.0)	0.39^b^
Dyslipidaemia	20 (40.8)	11 (45.8)	9 (36.0)	0.57^b^
Diabetes	15 (30.6)	8 (33.3)	7 (28.0)	0.76^b^
Creatinine (mg/dL, mean ± SD)	1.47 ± 1.11	1.31 ± 0.72	1.62 ± 1.37	0.32^c^
Renal failure (Cr > 1.5 mg/dL)	14 (28.6)	8 (33.3)	6 (24.0)	0.54^b^
COPD	2 (4.1)	0 (0.0)	2 (8.0)	0.49^d^
Cerebrovascular disease	3 (6.1)	1 (4.2)	2 (8.0)	>0.99^d^
Atrial fibrillation	4 (8.2)	2 (8.3)	2 (8.0)	>0.99^d^
Ejection fraction (%, mean ± SD)	47.4 ± 14.9	46.7 ± 15.2	48.0 ± 14.9	0.75^a^
EuroSCORE II (%, mean ± SD)	30.9 ± 14.1	29.9 ± 11.8	31.8 ± 16.2	0.65^c^
Duration from AMI to surgery (days, mean ± SD)	6.79 ± 8.57	1.58 ± 1.10	12.0 ± 9.60	<0.001^c^
Mechanical circulation support								
IABP	44 (89.8)	20 (83.3)	24 (96.0)	0.19^d^
ECMO	10 (20.4)	5 (20.8)	5 (20.0)	>0.99^d^
Cardiac arrest before operation	7 (14.3)	5 (20.8)	2 (8.0)	0.25^d^
Type of VSR				
Anterior/Apical	40 (81.6)	19 (79.2)	21 (84.0)	0.73^d^
Inferior/Posterior/Basal	9 (18.4)	5 (20.8)	4 (16.0)	0.73^d^
Free wall rupture	6 (12.2)	4 (16.6)	2 (8.0)	0.42^d^
Papillary muscle rupture	3 (6.1)	2 (8.3)	1 (4.0)	0.61^d^
VSR defect diameter (cm, mean ± SD)	2.26 ± 0.90	2.27 ± 0.75	2.25 ± 1.02	>0.99^c^
Left main trunk disease	3 (6.1)	1 (4.2)	2 (8.0)	>0.99^d^
Distal vessel disease				
Left anterior descending artery	42 (85.7)	19 (79.2)	23 (92.0)	0.25^d^
Left circumflex artery	15 (30.6)	9 (3.8)	6 (24.0)	0.36^b^
Right coronary artery	18 (36.7)	10 (41.7)	8 (32.0)	0.56^b^
Preoperative PCI	12 (24.5)	7 (29.2)	5 (20.0)	0.52^d^
Postoperative PCI	2 (4.1)	1 (4.2)	1 (4.0)	>0.99^d^
Surgical method				
Infarct exclusion	29 (59.2)	14 (58.3)	15 (60.0)	>0.99^b^
Extended sandwich patch technique	20 (40.8)	10 (41.7)	10 (40.0)	>0.99^b^
Intraoperative data				
Operation time (min, mean ± SD)	263.1 ± 86.9	251.4 ± 75.3	274.4 ± 96.8	0.36^c^
CPB time (min, mean ± SD)	168.5 ± 66.1	163.3 ± 53.3	173.5 ± 77.1	0.60^c^
Cross-clamp time (min, mean ± SD)	118.1 ± 46.3	110.7 ± 33.2	125.3 ± 55.9	0.26^c^
Concomitant procedures				
Coronary artery bypass grafting	28 (57.1)	12 (50.0)	16 (64.0)	0.39^b^
Mitral valve replacement	2 (4.1)	1 (4.2)	1 (4.0)	>0.99^d^
Mitral valve plasty	1 (2.0)	0 (0.0)	1 (4.0)	>0.99^d^
Tricuspid valve plasty	2 (4.1)	1 (4.2)	1 (4.0)	>0.99^d^

Early postoperative outcomes

The early postoperative outcomes are presented in Table 2. The 30-day mortality rates were 33.3% and 12.0% (P = 0.096), and the in-hospital mortality rates were 37.5% and 16.0% (P = 0.114) in groups A and B, respectively. Recurrent or residual shunt was observed in three (12.5%) and six (24.0%) patients in groups A and B, respectively (P = 0.463). Reoperation was performed in two patients in each group (P > 0.99). A postoperative IABP was used in 46 patients (93.9%), and the mean duration was 4.7 ± 3.3 days (P = 0.559). The comparison of other postoperative complications, including prolonged ventilation, postoperative stroke, re-exploration for bleeding, severe arrhythmia, postoperative atrial fibrillation, and use of renal replacement therapy, showed no significant differences between the two groups. However, tracheostomy was more common in group A (P = 0.05).

**Table 2:** Early postoperative outcomes Data are presented as n (%) unless otherwise indicated. P values less than 0.05 were considered statistically significant. ECMO, extracorporeal membrane oxygenation; IABP, intra-aortic balloon pump; ICU, intensive care unit; SD, standard deviation. *a* Determined by Student t-test, *b* Determined by Chi-square test, *c* Determined by Mann–Whitney U test, *d* Determined by Fisher's exact tests

	All n = 49	Group A n = 24	Group B n = 25	P-value
Mortality				
30 days	11 (22.4)	8 (33.3)	3 (12.0)	0.10^b^
In-hospital	13 (26.5)	9 (37.5)	4 (16.0)	0.11^b^
Complications				
Prolonged ventilation (>72 h)	39 (79.6)	19 (79.1)	20 (80.0)	>0.99^d^
Tracheostomy	4 (8.2)	4 (16.7)	0 (0.0)	0.05^ d^
Postoperative stroke	1 (2.0)	1 (4.2)	0 (0.0)	0.49^ d^
Reoperation	4 (8.2)	2 (8.3)	2 (8.0)	>0.99^ d^
Recurrent or residual shunt	9 (18.4)	3 (12.5)	6 (24.0)	0.46^ d^
Re-exploration for bleeding	3 (6.1)	3 (12.5)	0 (0.0)	0.11^ d^
Severe arrhythmia	5 (10.2)	2 (8.3)	3 (12.0)	>0.99^ d^
Postoperative atrial fibrillation	23 (46.9)	9 (37.5)	14 (56.0)	0.26^ d^
Renal replacement therapy	10 (20.4)	5 (20.8)	5 (20.0)	>0.99^ d^
Deep sternal wound infection	0 (0.0)	0 (0.0)	0 (0.0)	>0.99^ d^
Other postoperative data				
Intubation time (hours, mean ± SD)	140 ± 173.3	172.8 ± 229.0	108.6 ± 87.8	0.20^c^
ICU stay (days, mean ± SD)	10.7 ± 8.5	10.3 ± 9.3	11.0 ± 7.8	0.75^a^
Postoperative IABP	46 (93.9)	22 (91.7)	24 (96.0)	0.19^d^
Duration of IABP (days, mean ± SD)	4.7 ± 3.3	5.0 ± 4.0	4.44 ± 2.53	0.56^c^
Postoperative ECMO	4 (8.2)	3 (12.5)	1 (4.0)	0.35^d^

Table 3 presents the causes of in-hospital death. Low cardiac output syndrome was the most common cause of death, followed by bleeding, hypoxic-ischemic encephalopathy, multiple organ failure, and sepsis. Low cardiac output syndrome and bleeding were significantly common in group A (P = 0.023).

**Table 3:** Cause of in-hospital death ARDS, acute respiratory distress syndrome; DIC, disseminated intravascular coagulation; HIE, hypoxic-ischemic encephalopathy; LOS, low cardiac output syndrome; MOF, multiple organ failure; POD, postoperative day.

Patient number	POD	Age, years	Sex	Group A	Group B
1	1	75	Female	LOS, Bleeding, HIE	-
2	1	72	Male	LOS, Bleeding	-
3	1	67	Female	LOS, Bleeding	-
4	1	59	Male	-	LOS, HIE, MOF
5	3	73	Male	LOS, HIE	-
6	4	82	Female	LOS	-
7	5	85	Female	LOS, Bleeding	-
8	5	80	Female	Bleeding	-
9	11	85	Male	-	DIC, MOF
10	14	75	Female	-	HIE
11	15	82	Male	LOS, Sepsis	-
12	42	78	Female	Sepsis	-
13	67	84	Female	-	Sepsis, ARDS

Long-term clinical outcomes

**Figure 2:**
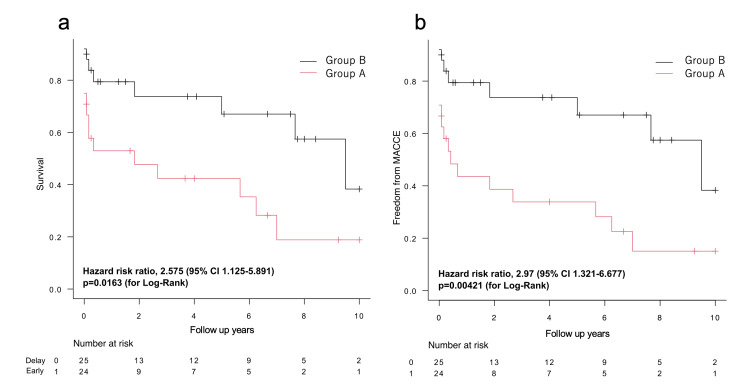
Kaplan–Meier estimates for freedom from all-cause death and major adverse cardiac and cerebrovascular events (MACCE) (a) Kaplan–Meier survival curve after ventricular septal rupture (VSR) repair, comparing group A (red line) and group B (black line). (b) Kaplan–Meier estimates for freedom from MACCE after VSR repair, comparing group A (red line) and group B (black line). CI, confidence interval.

The patients were followed up for 12 years, and the average follow-up time was 3.32 ± 3.68 years. Figure 2 shows the rates of survival and freedom from MACCE. The results were analyzed one, three, and 10 years after surgery. The overall survival curves estimated using the Kaplan-Meier method were 66.5 ± 6.9%, 58.2 ± 7.5%, and 28.8 ± 10.6% after one, three, and 10 years, respectively. The mortality in group B at 3 (P = 0.045) and 10 (P = 0.025) years was significantly better than that in group A. Regarding risk-adjusted outcomes, significant differences were observed between the three- (P = 0.045) and 10-year (P = 0.021) survival rates (Table 4). The rate of freedom from MACCE was significantly better in group B at all time points, and the results were similar after risk adjustment analysis.

**Table 4:** Cox regression analysis of survival and major adverse cardiac and cerebrovascular events in group A at one, three, and 10 years P values less than 0.05 were considered statistically significant. CI, confidence interval; HRR, hazard risk ratio; MACCE, major adverse cardiac and cerebrovascular events. *a* Data adjusted for EuroSCORE II, preoperative cardiopulmonary resuscitation, and preoperative extracorporeal membrane oxygenation.

		Crude			Adjusted^a^		
		HRR	95% CI	P-value	HRR	95% CI	P-value
Survival	1 year	2.664	0.923‒7.69	0.07	3.364	0.985‒11.48	0.05
	3 years	2.691	1.02‒7.097	0.05	2.984	1.025‒8.685	0.05
	10 years	2.575	1.125‒5.891	0.03	2.924	1.175‒7.273	0.02
MACCE	1 year	3.266	1.16‒9.195	0.03	3.542	1.141‒1.00	0.03
	3 years	3.249	1.256‒8.403	0.02	3.253	1.185‒8.930	0.02
	10 years	2.97	1.321‒6.677	0.009	3.195	1.331‒7.667	0.009

## Discussion

This study shows that surgery for VSR is associated with significantly improved long-term outcomes, all-cause mortality, and risk of MACCE when performed after 96 hours from the onset of myocardial infarction. Previous reports have shown the effectiveness of delayed surgery [1,3,12,13]; however, this study is the first to report long-term outcomes with a follow-up period of 10 years.

Since 2004, the American College of Cardiology/American Heart Association guidelines for ST-elevation myocardial infarction have recommended that all cases of VSR (including hemodynamically stable patients) be treated promptly. This is because patients may suddenly become hemodynamically unstable [14]. By contrast, the European Society of Cardiology guidelines (2017) consider delayed elective surgical repair in patients responding well to aggressive heart failure therapy [4], shifting the timing of surgical intervention for VSR from immediate to delayed.

In the current study, the two leading causes of in-hospital mortality in VSR were low cardiac output syndrome and bleeding. In order to restore the cardiac function lost as a result of AMI, repair involves incising the rupture or infarct site and suturing a patch over it that overlaps the non-infarcted myocardium; normal wall motion abnormalities are not expected in an area larger than the infarct site. This results in a decrease in ventricular volume and thus surgical intervention is associated with postoperative low cardiac output syndrome. Furthermore, surgery on fragile infarcted myocardium may induce hemostasis difficulties, promote bleeding, and lead to perioperative mortality.

The present results suggest that delaying VSR repair surgery by at least 96 hours after AMI onset improves postoperative outcomes. The progression of myocardial wound healing following infarction is divided into three stages: 1) an early inflammatory phase involving pronounced chemical signaling, necrotic tissue resorption, and myofibroblast recruitment; 2) a fibrotic phase involving increased myofibroblast numbers and collagen accumulation; and 3) a long-term remodeling phase involving collagen matrix stabilization and maturation [15,16]. The recruitment and activation of matrix metalloproteinases, which degrade collagen and other components of the myocardial extracellular matrix, can occur as early as 15 min post-AMI, with peak activation observed one to two days later [17,18]. Consequently, the infarct is mechanically the weakest and most prone to rupture during this period, when the degradation of the existing structure is underway, and significant deposition of new collagen has yet to begin [19]. Therefore, avoiding surgery during the early phase is reasonable. Delaying surgery for a few weeks after AMI may be necessary for ideal scar formation. However, the natural prognosis of the disease is poor. Delayed surgery with a mechanical circulatory support device is associated with the risks of bleeding, thrombosis, blood infection due to various lines, or being bedridden [3,13].

The limitations of this study include the relatively small sample size and single-center design, which may limit the generalizability of our results. In addition, the retrospective nature of the study introduces potential selection bias. The non-randomized design and the possibility of confounding factors may also influence the outcomes. For instance, the inherent variability in patient management and the potential differences in preoperative and postoperative care could affect the results. To address these limitations, future research could benefit from larger, multicenter studies with randomized controlled trials and detailed protocols to minimize bias. In addition, future studies should explore the impact of potential confounding factors and employ sensitivity analyses to validate the findings.

In summary, while our study provides valuable insights into the optimal timing of surgical intervention for VSR, the limitations highlight the need for further research to corroborate and expand upon these findings. Exploring these aspects in future studies will be essential for refining treatment strategies and improving patient outcomes.

## Conclusions

In this single-center retrospective study, VSR repair surgery >96 hours after the onset of AMI had better long-term postoperative mortality than those who underwent surgery within 96 hours. Furthermore, our study highlighted significant differences in MACCE between the two groups, with consistently lower incidence rates in the delayed surgery cohort across all follow-up periods.

These findings underscore the importance of considering optimal timing for VSR repair. The observed benefits of delayed surgery may be attributed to several factors, including improved myocardial healing and stabilization, reduced surgical complexity, and enhanced patient recovery. Our findings align with recent shifts in clinical guidelines, which advocate for a tailored approach to VSR management based on individual patient presentation and response to initial medical management.

However, the retrospective nature of this study and its single-center design may limit the generalizability of these results. While the data robustly supports the benefits of delayed surgery, the findings should be interpreted within the context of these design limitations.

To further validate these results and refine optimal timing strategies for VSR repair post-AMI, future research should focus on prospective, multicenter studies or randomized controlled trials. Such studies will be crucial in confirming the advantages of delayed surgery and addressing any potential biases inherent in retrospective analyses.

## References

[1] Shafiei I, Jannati F, Jannati M (2020). Optimal time repair of ventricular septal rupture post myocardial infarction. J Saudi Heart Assoc.

[2] Matteucci M, Ronco D, Corazzari C (2021). Surgical repair of postinfarction ventricular septal rupture: systematic review and meta-analysis. Ann Thorac Surg.

[3] Żbikowska K, Wróbel K (2022). Mechanical circulatory support in delayed surgery of post-infarction ventricular septal rupture in patients in cardiogenic shock-a review. J Clin Med.

[4] Ibanez B, James S, Agewall S (2018). 2017 ESC Guidelines for the management of acute myocardial infarction in patients presenting with ST-segment elevation: The Task Force for the management of acute myocardial infarction in patients presenting with ST-segment elevation of the European Society of Cardiology (ESC). Eur Heart J.

[5] Dallan LR, Dallan LA, Lisboa LA (2021). Increased number of ventricular septal rupture cases after acute myocardial infarction in 2020. J Card Surg.

[6] Sánchez Vega JD, Alonso Salinas GL, Viéitez Florez JM (2022). Optimal surgical timing after post-infarction ventricular septal rupture. Cardiol J.

[7] David TE, Dale L, Sun Z (1995). Postinfarction ventricular septal rupture: repair by endocardial patch with infarct exclusion. J Thorac Cardiovasc Surg.

[8] Asai T, Hosoba S, Suzuki T, Kinoshita T (2012). Postinfarction ventricular septal defect: right ventricular approach-the extended "sandwich" patch. Semin Thorac Cardiovasc Surg.

[9] Kinoshita T, Asai T, Hachiro K, Suzuki T (2022). Extended sandwich patch technique via right ventriculotomy for acute ventricular septal rupture. Ann Thorac Surg.

[10] Sugimoto T, Yoshii S, Yamamoto K (2008). A modified infarct exclusion technique: triple-patch technique for postinfarction ventricular septal perforation. J Thorac Cardiovasc Surg.

[11] Nashef SA, Roques F, Sharples LD, Nilsson J, Smith C, Goldstone AR, Lockowandt U (2012). EuroSCORE II. Eur J Cardiothorac Surg.

[12] Arnaoutakis GJ, Zhao Y, George TJ, Sciortino CM, McCarthy PM, Conte JV (2012). Surgical repair of ventricular septal defect after myocardial infarction: outcomes from the Society of Thoracic Surgeons National Database. Ann Thorac Surg.

[13] Furui M, Sakurai Y, Kakii B, Asanuma M, Nishioka H, Yoshida T (2022). Benefits and risks of delayed surgery for ventricular septal rupture after acute myocardial infarction. Int Heart J.

[14] Antman EM, Anbe DT, Armstrong PW (2004). ACC/AHA guidelines for the management of patients with ST-elevation myocardial infarction--executive summary: a report of the American College of Cardiology/American Heart Association Task Force on Practice Guidelines (Writing Committee to Revise the 1999 Guidelines for the Management of Patients With Acute Myocardial Infarction). Circulation.

[15] Dobaczewski M, Gonzalez-Quesada C, Frangogiannis NG (2010). The extracellular matrix as a modulator of the inflammatory and reparative response following myocardial infarction. J Mol Cell Cardiol.

[16] Holmes JW, Borg TK, Covell JW (2005). Structure and mechanics of healing myocardial infarcts. Annu Rev Biomed Eng.

[17] Pfeffer MA, Braunwald E (1990). Ventricular remodeling after myocardial infarction. Experimental observations and clinical implications. Circulation.

[18] Takahashi S, Barry AC, Factor SM (1990). Collagen degradation in ischaemic rat hearts. Biochem J.

[19] Richardson WJ, Clarke SA, Quinn TA, Holmes JW (2015). Physiological implications of myocardial scar structure. Compr Physiol.

